# Impact of Antibiotic-Induced Depletion of Gut Microbiota and Selenium Supplementation on Plasma Selenoproteome and Metal Homeostasis in a Mice Model

**DOI:** 10.1021/acs.jafc.1c02622

**Published:** 2021-06-25

**Authors:** Belén Callejón-Leblic, Marta Selma-Royo, María Carmen Collado, Nieves Abril, Tamara García-Barrera

**Affiliations:** † Research Center of Natural Resources, Health and the Environment (RENSMA), Department of Chemistry, Faculty of Experimental Sciences, University of Huelva, Fuerzas Armadas Avenue, 21007 Huelva, Spain; ‡ Department of Biotechnology, Institute of Agrochemistry and Food Technology-National Research Council (IATA-CSIC), Agustin Escardino 7, Paterna, 46980 Valencia, Spain; § Department of Biochemistry and Molecular Biology, University of Córdoba, Campus de Rabanales, Edificio Severo Ochoa, E-14071 Córdoba, Spain

**Keywords:** selenoproteins, microbiota, chemical speciation, heteroatom-tagged proteomics, ICP-MS

## Abstract

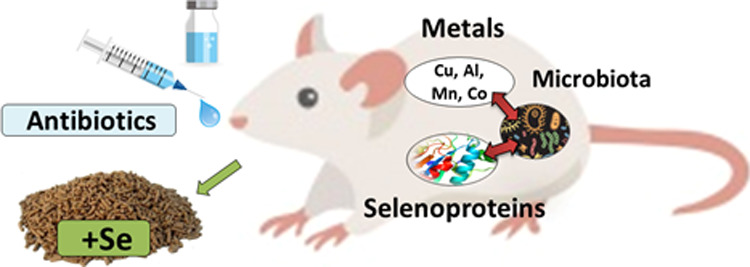

Selenium (Se) is a micronutrient involved in important health functions and it has been suggested to shape gut microbiota. Limited information on Se assimilation by gut microbes and the possible link with selenoproteins are available. For this purpose, conventional and gut microbiota-depleted BALB/c mice were fed a Se-supplemented diet. The absolute quantification of mice plasma selenoproteins was performed for the first time using heteroatom-tagged proteomics. The gut microbiota profile was analyzed by 16S rRNA gene sequencing. Se-supplementation modulated the concentration of the antioxidant glutathione peroxidase and the Se-transporter selenoalbumin as well as the metal homeostasis, being influenced by microbiota disruption, which suggests an intertwined mechanism. Se also modulated microbiota diversity and richness and increased the relative abundance of some health-relevant taxa (*e.g.*, families *Christensenellaceae*, *Ruminococcaceae*, and *Lactobacillus* genus). This study demonstrated the potential beneficial effects of Se on gut microbiota, especially after antibiotic-treatment and the first associations between specific bacteria and plasma selenoproteins.

## Introduction

The role of selenium (Se) in biology has been extensively reviewed due to its antioxidant character and the potential relevance to certain diseases such as cancer^1^ or cardiovascular disease.^2^ Thus, there is great interest in developing Se-enriched functional foods and nutraceuticals.^3^ The main source of Se is the diet, but the relationship between the status and dietary intake of this micronutrient is close to a U-shape, where adverse effects are derived from deficiency and excess, the Se-essentiality being conditioned to a narrow range of concentrations.^4^ This means that Se-enriched nutraceuticals and functional foods should control the bioavailable concentration of this element; however, the chemical form of Se used is also of importance.^5^ The most commonly marketed Se-enriched product is yeast *Saccharomyces cerevisiae*, but other functional foods have also been proposed such as *Chlorella sorokiniana*.^5^ Moreover, minerals such as Se can shape the colonization of gut microbiota, deeply affecting the host health.^6^ Accumulating data are demonstrating the pivotal role of gut microbiota on human health. Gut dysbiosis has been associated with high risk of metabolic and inflammatory alterations.^7^ Gut microbiota, in turn, can act as a barrier or modulator for nutrients, toxins, and pollutants.^8^ Nowadays, there is growing interest in the design of dietary strategies for the modulation and the re-building of microbiota.^9^ Few works have reported the impact of Se-supplemented diet gut microbiota because most of them have only focused on a Se-deficient diet.^10,11^ Zhai *et al.* concluded that supranutritional Se intake in the form of Na_2_SeO_3_ can optimize the gut microbiota for protection against intestinal dysfunctions in specific pathogen-free mice,^8^ and Liu *et al.* reported a partial restoring of the abundance of gut flora after Se-treatment of rats exposed to methylmercury.^12^ Although the beneficial functions of Se for gut microbiota have been attributed to selenoproteins and selenometabolites,^8^ little is known about the effect of Se-supplementation on host plasma selenoproteome and the potential link with gut microbiota. Similarly, the absolute quantification of plasma selenoproteins and correlation with specific bacteria have not been reported before and few works determined the expression profiles of certain selenoproteins after Se-supplementation in conventional (CV) and germ-free (GF) mice by enzymatic activities^10^ or western blot complemented with quantitative polymerase chain reaction (PCR).^13^

In this sense, the aim of this study was the absolute quantification, by the first time, of plasma selenoproteins in Se-supplemented CV and mice with microbiota depleted by antibiotics as well as their associations with specific bacteria. Selenoproteins have been determined using a highly sensitive and selective analytical technique, namely, heteroatom-tagged proteomics and the gut microbiota taxonomy by 16S rRNA gene sequencing. The impact of Se-supplementation on gut microbiota diversity, richness, and composition has been determined in both mice models. We also studied the influence of Se-supplementation and gut microbiota disruption in metal homeostasis and established the correlations between their concentration and the relative abundance of specific bacteria.

## Materials and Methods

### Animals, Experimental Design, and Dosage Information

**Figure 1:**
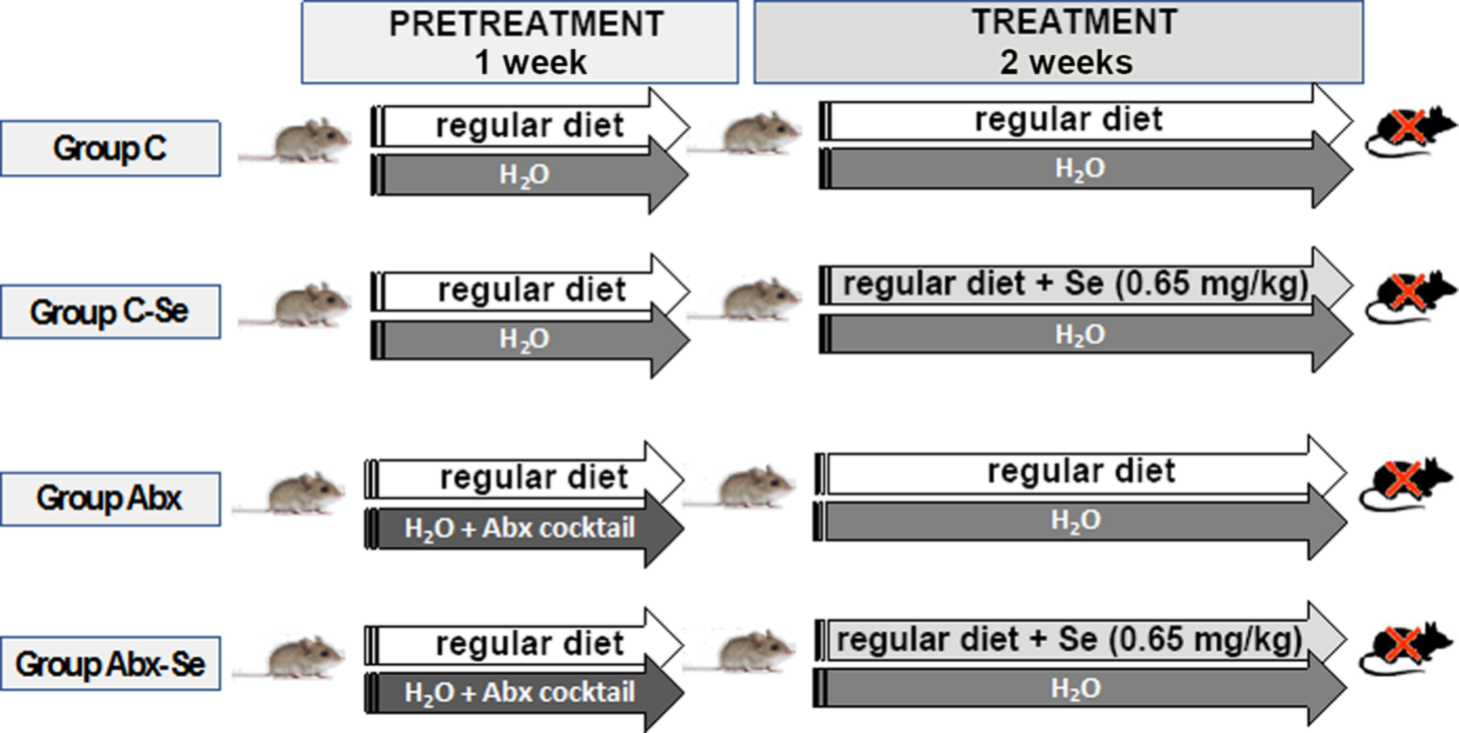
Experimental design showing the studied groups.

Male *Mus musculus* mice (inbred BALB/c strain, 8 weeks, 23–25 g) were purchased from Charles River Laboratories (Spain). The experiments were carried out in the Animal Experimentation Service of the University of Cordoba (SAEX-UCO) in a conditioned laboratory with controlled temperature (25 ± 2 °C) and photoperiod (12:12 h). The mice had free access to food and water, which were changed every second day to maintain their quality and weighed to calculate the actual ingested doses of experimental compounds. Forty mice were randomly divided into four groups (10 mice per group). The reference group (group C) was fed a rodent diet for 3 weeks (around 0.20 mg Se kg^–1^ chow). The group C–Se was fed the regular rodent diet for a week and then a Se-enriched diet containing 0.65 mg Se kg^–1^ chow as sodium selenite for the last two additional weeks. This non-toxic Se concentration was selected according to literature^14,15^ and our previous works about the influence of Se in mice metabolism and its antagonistic action against toxic compounds.^16,17^ Mice in the Abx and Abx-Se groups received the regular diet and water containing a cocktail of broad-spectrum antibiotics (ampicillin 1 g L^–1^, neomycin 1 g L^–1^, metronidazole 1 g L^–1^, vancomycin 0.5 g L^–1^, and the antifungal amphotericin B 10 mg L^–1^) during the first week. They were fed the regular diet for 3 weeks (Abx) or for 1 week followed by the Se-supplemented diet for the two additional weeks (Abx-Se). The selection of this cocktail was also based on the literature.^18−20^Figure 1 shows the experimental design of the study. At the end of the experimental time, mice were anesthetized by isoflurane inhalation, exsanguinated by cardiac puncture, and dissected using a ceramic scalpel. All animals received humane care in compliance with animal care guidelines and use of the European Community. The investigation was performed with the consent of the Ethical Committee of the University of Córdoba (Spain) (code num. 02-01-2019-001).

### Biological Samples

Blood samples were collected in heparinized tubes that were centrifuged (3000*g*, 10 min, room temperature) within 30 minutes after blood collection to obtain the plasma. Large intestinal content was collected and flash-frozen in liquid nitrogen. Both plasma aliquots and gut samples were stored at −80 °C until analysis.

### Antibiotic Cocktail, Standard Solutions, and Reagents

Ammonium acetate (NH_4_CH_3_CO_2_), sodium selenite (Na_2_SeO_3_), the antibiotics ampicillin, neomycin, metronidazole vancomycin, and the antifungal amphotericin B were purchased from Sigma-Aldrich, (Steinheim, Germany). Trace metal grade nitric acid (HNO_3_) was obtained from Fisher Scientific (Leicestershire, UK). Enriched ^74^Se for isotopic dilution analysis was obtained from Cambridge Isotope Laboratories (Andover, MA). The BCR-637 human serum certified reference material (CRM) was purchased from the Institute for Reference Materials and Measurements (IRMM, Geel, Belgium). Serum Control for Trace Element lyophilized for Trace Elements, Level II was obtained from ClinChek, RECIPE (Munich, Germany). Water was purified with a Milli-Q Gradient system (Millipore, Watford, UK). A DNA Purification Kit was obtained from Macherey–Nagel (Duren, Germany), a Master-Pure DNA extraction Kit from Epicentre (Madison, WI, US), and a NextEra Index Kit from Illumina (San Diego, CA, United States).

### Speciation of Selenoproteins in Mice Plasma

Speciation of selenoproteins in plasma from mice was carried out by a column switching method coupled to an inductively coupled plasma mass spectrometer as described previously.^21^ Briefly, before the analysis, plasma samples were filtered using Iso-Disc filters of polyvinylidene difluoride (PVDF) (20 mm of diameter and 0.45 μm of pore size). Then, 100 μL of plasma was injected into a high-performance liquid chromatograph model 1260 Infinity Quaternary LC (Agilent Technologies) connected to two 5 mL HiTrap Desalting Columns (GE Healthcare, Uppsala, Sweden) and two affinity columns of heparin-sepharose (HEP-HP) and blue-sepharose (BLU-HP) (GE Healthcare, Uppsala, Sweden). Ammonium acetate was used for the preparation of mobile phases A (0.05 M, pH = 7.4) and B (1.5 M, pH = 7.4) and the flow-rate was set at 1.3 mL min^–1^. The columns were interconnected using a six-way valve and finally, they were coupled to a triple quadrupole inductively coupled plasma mass spectrometer model Agilent 8800 Triple Quad (Agilent Technologies, Tokyo, Japan) through a Micromist nebulizer (Glass Expansion, Switzerland). The HEP-HP column is able to retain selenoprotein P (SEPP1), while the BLU-HP column retains both SEPP1 and selenoalbumin (SeAlb). To separate the selenoproteins, we applied two working modes: (i) Mode 1 (from 0 to 20 min, mobile phase A); the plasma sample passes through the whole system 2D-SEC-SEC-AF(HEP-HP)xAF(BLU-HP)-ICP-MS, allowing the elution of plasma glutathione peroxidase (GPx) and selenometabolites at 4 and 8 min, respectively, and the retention of SEPP1 in the HEP-HP column and SeAlb in BLU-HP column; (ii) Mode 2 (from 20 to 24 min, mobile phase B); SEPP1 elutes at 20.5 min and SeAlb is isolated in the BLU-HP column; (iii) Mode 3 (from 24 to 40 min, mobile phase B); SeAlb is released and can elute at 25 min. The absolute quantification of selenocompounds was carried out using the species unspecific isotopic dilution analysis. To this end, a flow-rate of 0.1 mL min^–1^ of Se-enriched standard (^74^Se Cambridge Isotope Laboratories, Andover, MA, USA) was introduced into the system after the chromatographic separation (post-column) using a T shape connector. The instrumental conditions for the speciation of selenoproteins have been previously described.^21^ The quality of the analytical method (Table S1) was verified using the human serum BCR-637 CRM (Institute for Reference Materials and Measurements, IRMM, Geel, Belgium).

### Total Determination of Elements in Mice Plasma

Total elemental analysis of Al, V, Cr, Mn, Fe, Co, Ni, Cu, Zn, As, Se, Mo, Cd, Sb, Tl, and Pb was performed on an Agilent 8800 triple quadrupole inductively coupled plasma mass spectrometer. Before the analysis, plasma samples were fivefold diluted with water and filtered using PVDF filters. For the quantification of the majority of elements (except for Mo and Sb), a multi-element calibration standard solution (10 mg L^–1^, Agilent Technologies) was used to prepare the calibration curves from 0 to 250 ng g^–1^. Individual standard solutions of Mo and Sb were necessary to determine the concentration of these elements in plasma samples. In addition, 0.1 mg L^–1^ of rhodium was used as the internal standard. A serum control (Trace Element, Level II, RECIPE) was treated and analyzed with the same conditions as samples to check the variability and reproducibility of the analysis (Table S2). Instrumental conditions for the analysis are also described in the Supporting Information.

### Determination of the Gut Microbiota Profile in Mice

Total DNA was extracted from the frozen fecal material (approx. 100 mg) using a MasterPure the DNA extraction Kit (Epicentre, Madison, WI, US) following the manufacturer’s instructions with the following modifications: samples were treated with lysozyme (20 mg mL^–1^) and mutanolysin (5 U mL^–1^) for 60 min at 37 °C and a preliminary step of cell disruption with 3 μm diameter glass beads during 1 min at 6 m s^–1^ by a bead beater FastPrep 24-5G Homogenizer (MP Biomedicals) as described elsewhere.^22^ Purification of the DNA was performed using a DNA Purification Kit (Macherey–Nagel, Duren, Germany) according to the manufacturer’s instructions and the DNA concentration was measured using a Qubit 2.0 Fluorometer (Life Technology, Carlsbad, CA, US) for further analysis.

The gut microbiota profile was determined by the V3–V4 variable region of the 16S rRNA gene sequencing following Illumina protocols. Briefly, a multiplexing step was conducted using a NextEra Index Kit (Illumina, San Diego, CA, United States) and amplicons were checked with a Bioanalyzer DNA 1000 chip (Agilent Technologies, Santa Clara, CA, United States). Libraries were sequenced using a 2 × 300 bp paired-end run (MiSeq Reagent kit v3) on a MiSeq-Illumina platform (FISABIO sequencing service, Valencia, Spain) according to manufacturer’s instructions. Controls during DNA extraction and PCR amplification were also included and sequenced. Residual adaptors were removed from the raw sequences by the use of Trimmomatic software.^23^ A DADA2 pipeline was used to achieve quality filtering, sequence joining, and chimera removal.^24^ Taxonomic assignment, including the specie level classification, was performed by using the Silva v132 database.^25,26^ Samples with less than 1000 reads were removed from the study. Taxa present in a relative abundance less than 0.01% and those present in less than 3 times in at least 20% of the samples were also filtered. Furthermore, sequences classified as Cyanobacteria and Chloroplast were removed from the final data set as they represent potential contaminants.

### Statistical Analysis

One-way ANOVA and a Tukey test for multiple comparisons were applied to the results using STATISTICA 8.0 from StatSoft. Spearman correlations between selenoproteins, metals, and microbiota (phylum and genus levels) were determined using R Software Package Hmisc (4.0.2 version).^27^ For the microbiota analyses, Calypso web platform v. 8.56^28^ was used with total sum normalization for the statistical analysis, multivariate test, and data mining. Alpha-diversity metrics (Chao1 and Shannon indexes) were obtained at the amplicon sequence variant (ASV) level after rarefaction to the minimum reads number (93,525 reads). Permutational multivariate ANOVA using Bray–Curtis distance (Adonis) at the ASV level was performed and the visualization of the multivariate analysis was assessed by redundancy discriminant analysis (RDA). Data were classified by metadata factors and differences in relative abundance were evaluated by the Wilcoxon test with false discovery test rate (FDR) for multiple test corrections. Comparisons 2 × 2 of microbiota composition at the phylum and genus levels were performed by the DESeq2 approach with the false discovery rate correction. The level of statistical significance for all tests was fixed at *p* < 0.05.

## Results and Discussion

Herein, we report the impact/effect of a Se-enriched diet on selenoproteins and total Se in mice plasma in the presence and absence of antibiotics to induce gut microbiota depletion. The estimated daily ingestion of Se was about 40 μg kg^–1^ bw for mice fed the regular diet and about 120 μg kg^–1^ bw for the mice fed the Se-enriched diet. Popular Se supplement products, including both organic and inorganic chemical forms, usually do not exceed 200 μg/day (about 3 times the requirement)^29^ to avoid the inhibitory or toxic effect exerted by Se at a high dose.^30^ Since the regular mouse chow diet in our study contains about 0.20 mg Se kg^–1^ chow, we decided to formulate a Se-enriched diet containing 0.65 mg Se kg^–1^ chow (about 3 times the regular Se ingestion). As previously reported,^20^ we selected a cocktail containing the antibiotics ampicillin, neomycin, metronidazole, vancomycin, and the antifungal amphotericin B to deplete the gut microbiota. The estimated daily ingestion of antibiotics during the pretreatment by mice in Abx and Abx-Se groups was 200 mg kg^–1^ bw of ampicillin, neomycin, and metronidazole; 100 mg kg^–1^ bw of vancomycin; and 2 mg kg^–1^ bw of amphotericin B. No lethality was observed during the different phases of treatment, but the antibiotics pretreatment caused a severe weight lost in mice, which was quickly recovered after moving to the treatment phase.

### Impact of Selenium Supplementation on Plasma Selenoproteome Is Affected by Microbial Antibiotic Disruption

Quantification of plasma selenoproteins and selenometabolites was performed by unspecific isotopic dilution analysis using the chromatographic column switching method 2D-SEC-SEC-AF(HEP-HP)xAF(BLU-HP)-ICP-MS described previously. This analytical method allows the absolute quantification of selenoproteins using a heteroelement (an atom different to C, H, N, O, or F, *e.g.*, Se) of the biomolecule as a “tag” in a sensitive and selective detector such as ICP–MS.^31^ Thus, using heteroatom-tagged proteomics, the absolute concentration of selenoproteins (as Se) can be determined instead of the enzymatic activity or their relative concentration typically used in protein analysis.

**Figure 2:**
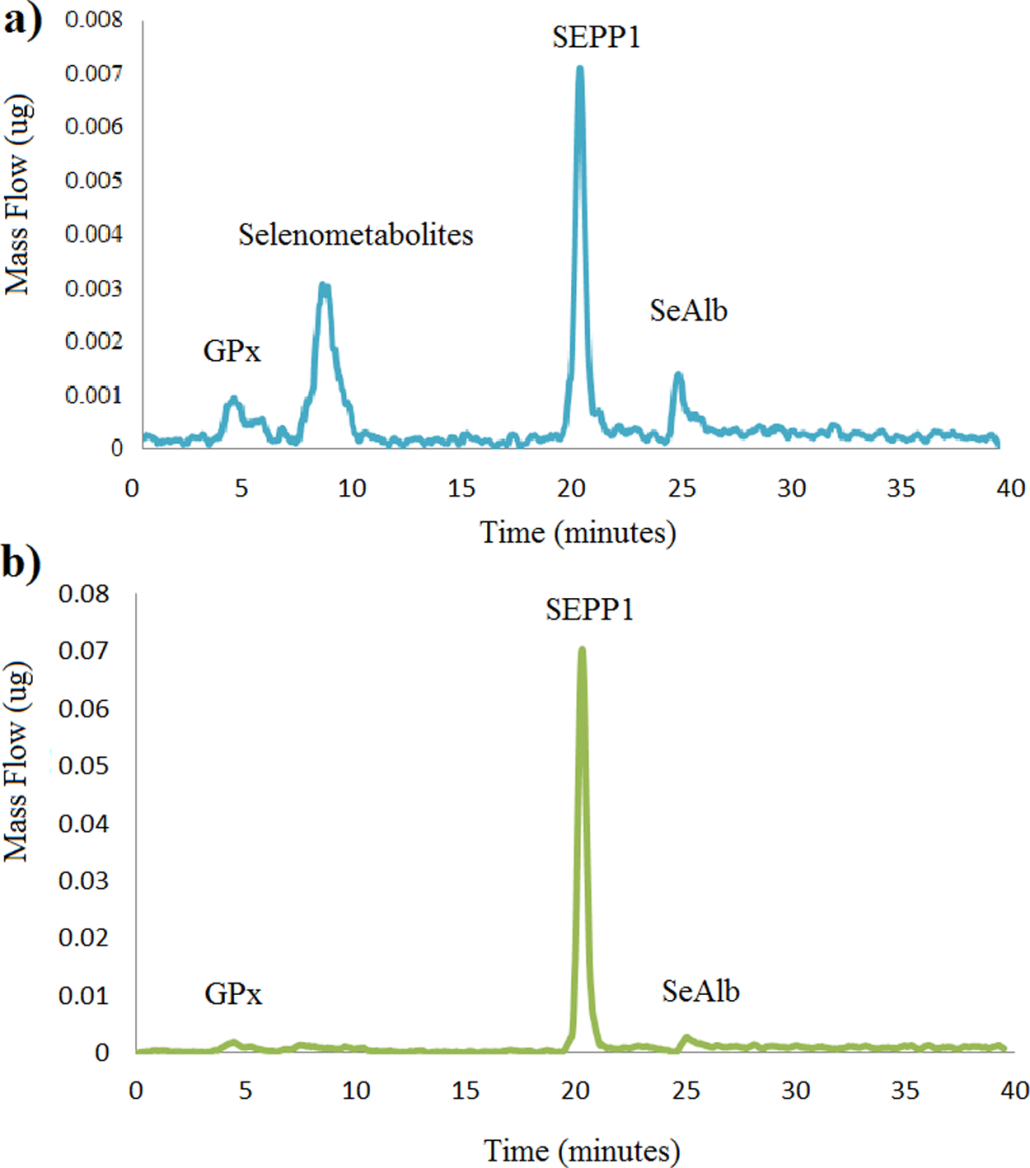
Mass flow chromatograms corresponding to (a) BCR-637 fortified with 50 ng g^–1^ of selenite and (b) plasma mice after speciation of selenoproteins.

Figure 2 shows the mass flow chromatograms of (a) the BCR-637 serum CRM spiked with 50 ng g^–1^ of sodium selenite and (b) a mice plasma sample. Levels of selenometabolites (which elute in a single peak after GPx) were lower than the detection limit in all the samples. In the bloodstream, SEPP1 accounts for >50% of Se, followed by SeAlb (∼15–20%) and GPx (∼15–20%),^32^ which is in good agreement with our results. These three selenoproteins are the most commonly used markers for the assessment of Se status in human plasma/serum.^33^ To the best of our knowledge, SeAlb has not been previously reported in microbiota studies after Se-supplementation. SeAlb transports Se to the liver for the production of the majority of selenoproteins and delivery of metabolites to the plasma.^34^

**Figure 3:**
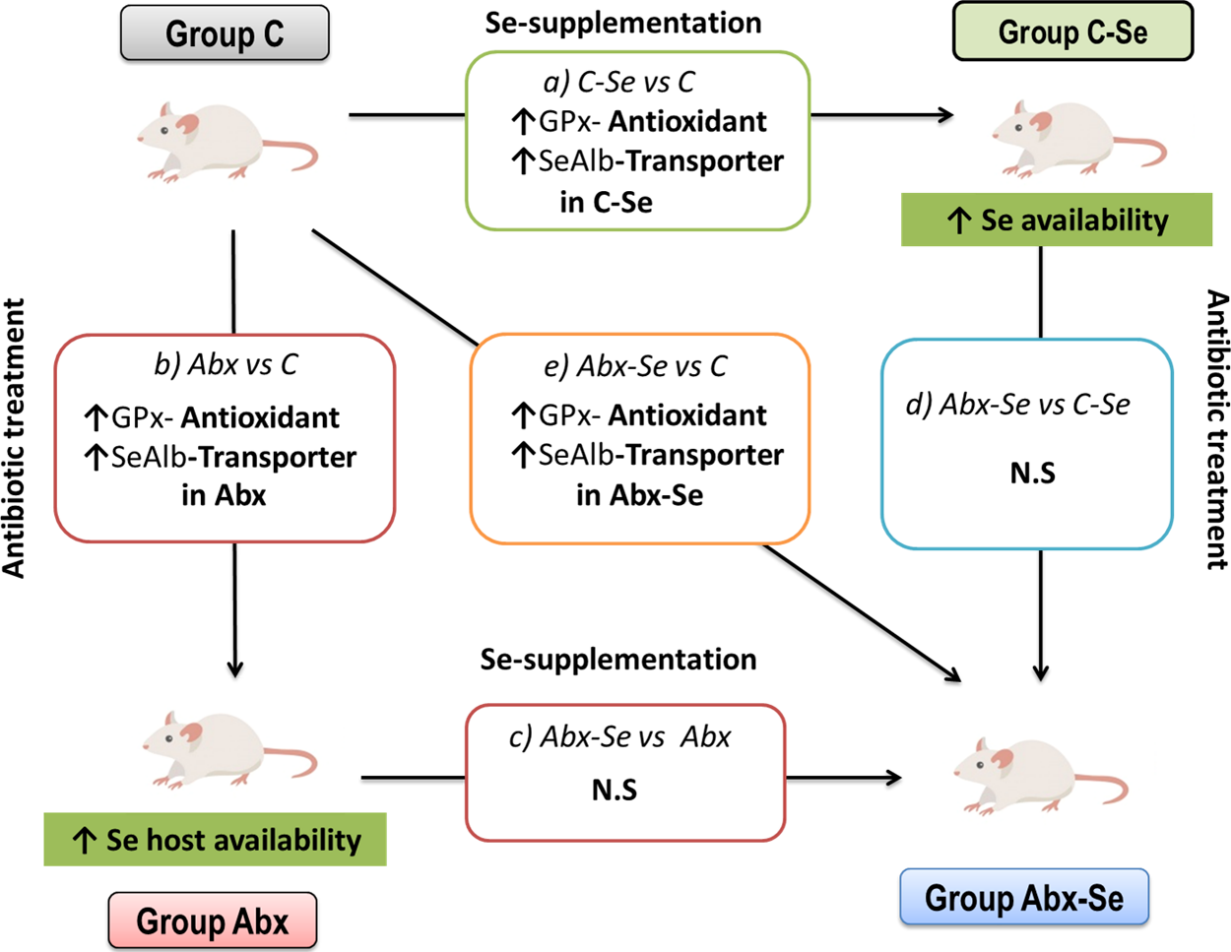
Model map showing the mechanism underlying the potential beneficial effects of Se in the conditions with and without antibiotics.

A one-way ANOVA was carried out to determine the statistical significance of the differences observed among the four experimental groups C, C–Se, Abx, and Abx-Se regarding both the total Se and plasma selenoprotein concentrations (Table 1). The average concentration of SEPP1 and total Se did not change significantly when comparing the groups under study, suggesting that the main role of SEPP1 (*i.e.*, the transport of Se from the liver to other organs or prevention of neurotoxicity^35^) did not result in being altered either by Se-supplementation or antibiotics-induced microbiota depletion at the studied levels. However, as commented in the next sections, this protein and total Se correlate with specific bacteria in the different groups, showing their interplay with gut microbiota. In contrast, the ANOVA showed significant increases of GPx and SeAlb levels after Se-supplementation of CV mice diet (groups C–Se *vs* C). The increases in GPx and SeAlb levels were also significant after microbiota depletion (Abx *vs* C) and when analyzing the combined effect of Se-supplementation and microbiota depletion (Abx-Se *vs* C) (Table 1). No significant changes in GPx and SeAlb abundances were observed in the comparisons between Abx-Se versus Abx or Abx-Se versus C–Se. Thus, Se-supplementation affected the GPx concentration in both CV and Abx, indicating an increase in the antioxidant function of the host.^36^ The reason for the increase in the levels of GPx and SeAlb after depletion of the microbiota by antibiotics (Abx *vs* C) is less obvious. It has been reported that about one-quarter of all bacteria express selenoproteins and therefore sequester some Se for optimal growth and their normal metabolic functions.^13^ The explanation could be that the bacteria that grow after antibiotic treatment are less able to sequester Se, thus decreasing competition with the host. Then, a higher Se availability would lead to higher levels of GPx and SeAlb in the Abx mice. Figure 3 shows a model map of the mechanism underlying the potential beneficial effects of Se in the conditions with and without antibiotics. In agreement with our data, it has been also showed that GF mice fed Se diets had an expression profile of certain selenoproteins similar to control mice but showed higher activity of GPx and methionine-R-sulfoxide reductase 1 in the liver, suggesting the partial use of Se by the gut microbes.^13^

**Table 1:** Average Concentration of Selenium in Selenoproteins, Total Selenium, and Fold Changes^a^

selenoproteins					
groups	GPx	selenometabolites	SEPP1	SeAlb	total Se
Concentration (ng of Selenium per g of Plasma) ± S.E.M (*n* = 10 Mice per Group)					
C	15.4 ± 1.8	<LOD	381.1 ± 11.7	27.8 ± 2.4	434.5 ± 14.3
C–Se	28.3 ± 2.3	<LOD	414.0 ± 22.0	49.3 ± 5.7	509.0 ± 28.6
Abx	22.3 ± 1.1	<LOD	401.2 ± 17.7	50.7 ± 3.0	483.2 ± 17.7
Abx-Se	28.0 ± 1.5	<LOD	398.6 ± 18.3	46.8 ± 4.1	489.3 ± 18.8
Fold Change					
C–Se/C	1.84 (*p* < 0.001)		1.09	1.77 (*p* < 0.003)	1.16
Abx/C	1.44 (*p* < 0.04)		1.05	1.82 (*p* < 0.002)	1.12
Abx-Se/C	1.82 (*p* < 0.001)		1.05	1.68 (*p* < 0.01)	1.12
Abx-Se/Abx	1.26		0.99	0.92	1.00

a LOD: detection limit of selenometabolites 0.5 ng Se g^–1^; *p*: *p*-value from ANOVA followed by Tukey Test (only significant *p*-values are shown in the table). *p* < 0.05 was considered statistically significant.

### Impact of the Selenium Supplementation and Microbiota Depletion on Trace Elements Homeostasis Is Affected by Microbial Antibiotic Disruption

The concentrations of several metals and metalloids (Al, V, Cr, Mn, Fe, Co, Ni, Cu, Zn, As, Mo, Cd, Sb, Tl, and Pb) determined by triple quadrupole inductively coupled plasma mass spectrometry (ICP-QQQ-MS) on mice plasma from the different groups studied in this work (C, C–Se, Abx and Abx-Se) are shown in Table 2. Fold changes between groups are also listed in Table 2 and only significant *p*-values are shown. The homeostasis of elements has a key importance on human health since numerous antagonistic and synergistic interactions between elements have been described in the literature.^37^ In fact, Se is a well-known antagonist against a great number of pollutants, including mercury, arsenic, and organic compounds.^37^ However, few studies have described metal homeostasis in mice after Se-supplementation^38,39^ and only Kasaikina *et al.* have reported the influence of Se status and gut microbes on other elements in GF mice organs.^13^ These authors only found higher levels of Cd in the liver from GF mice, suggesting a possible antagonistic role of the gastrointestinal microbiota against this element.^13^ Thus, this is the first time that statistically significant differences have been found in the plasma multielemental profile after Se supplementation, especially in mice with microbiota depleted by antibiotics. Se supplementation increased the levels of Al and Mo in plasma from CV mice (C–Se *vs* C) and Zn in mice with depletion of microbiota by antibiotics (Abx-Se *vs* Abx). Remarkably, most of the differences were found in microbiota-depleted mice fed a Se-supplemented diet (Abx-Se *vs* C–Se), which may indicate that in the absence of microbiota, the influence of Se in metal homeostasis is completely different. Thus, the concentrations of Al, V, Cu, and Co were significantly lower in Abx-Se against C–Se, while the concentration of Co diminished and Zn increased significantly against C (Abx-Se *vs* C). These results may indicate that metal homeostasis is affected by Se-supplementation and could be linked with gut microbiota, as significant differences were observed between CV and antibiotic-depleted microbiota groups. This is in good accordance with the results previously discussed about the influence of Se-supplementation on the plasma selenoproteome.

**Table 2:** Metal Profile in Mice Plasma^a^

	average concentration ± S.E.M				fold changes			
elements	C	C–Se	Abx	Abx-Se	C–Se/C	Abx-Se/C	Abx-Se/C–Se	Abx-Se/Abx
Al	25.4 ± 2.9	39.0 ± 4.3	24.2 ± 2.9	23.4 ± 2.7	1.54 (*p* = 0.03)	0.92	0.60 (*p* = 0.01)	0.97
V	10.4 ± 0.8	13.6 ± 1.4	9.7 ± 0.7	9.1 ± 1.1	1.31	0.87	0.67 (*p* = 0.03)	0.94
Cr	7.6 ± 0.7	11.1 ± 1.7	7.7 ± 0.5	7.3 ± 1.1	1.45	0.95	0.66	0.94
Mn	5.6 ± 0.5	8.2 ± 1.8	7.3 ± 1.1	7.4 ± 1.9	1.45	1.32	0.91	1.02
Fe	7448 ± 1264	6504 ± 1412	6099 ± 946	6108 ± 937	0.87	0.82	0.94	1.00
Co	6.2 ± 0.4	6.2 ± 0.5	5.3 ± 0.4	4.7 ± 0.5	1.01	0.76 (*p* = 0.01)	0.75 (*p* = 0.04)	0.89
Ni	4.5 ± 0.9	6.8 ± 1.3	3.2 ± 0.4	4.2 ± 0.9	1.51	0.92	0.61	1.28
Cu	649 ± 24	684 ± 33	602 ± 15	584 ± 29	1.05	0.90	0.85 (*p* = 0.05)	0.97
Zn	1031 ± 40	1071 ± 38	1014 ± 46	1213 ± 74	1.04	1.18 (*p* = 0.04)	1.13	1.20 (*p* = 0.03)
As	23.3 ± 3.6	22.2 ± 4.1	20.3 ± 3.0	19.7 ± 2.5	0.95	0.84	0.89	0.97
Mo	29.6 ± 1.6	45.4 ± 5.4	39.8 ± 7.5	33.4 ± 2.9	1.53 (*p* = 0.03)	1.13	0.74	0.84
Cd	0.12 ± 0.04	0.03 ± 0.01	0.09 ± 0.03	0.02 ± 0.01	0.22	0.16	0.74	0.20
Sb	6.7 ± 0.5	9.2 ± 1.1	6.1 ± 0.5	6.7 ± 0.5	1.36	1.00	0.73	1.09
Tl	0.51 ± 0.08	0.70 ± 0.12	0.49 ± 0.08	0.37 ± 0.10	1.37	0.72	0.52	0.74
Pb	2.2 ± 0.2	2.9 ± 0.6	2.7 ± 0.6	2.3 ± 0.4	1.34	1.03	0.77	0.85

a Concentrations, fold changes, *p*-values from ANOVA (only significant *p*-values are shown in the table), and standard error of the mean (S.E.M) of the elements. *p* < 0.05 was considered statistically significant.

### Impact of Antibiotic and Selenium Supplementation on the Gut Microbiota

**Figure 4:**
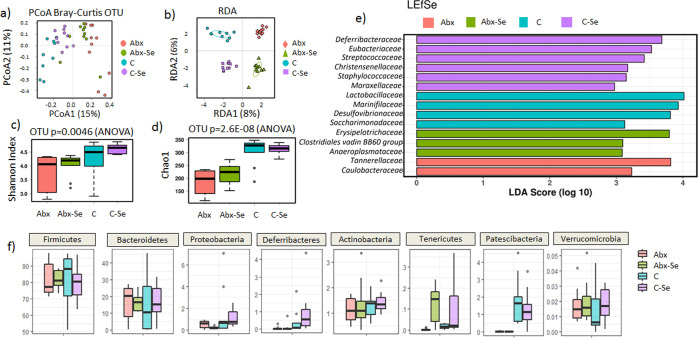
Impact of Se-supplementation on the microbiota of control mice and microbiota-depleted mice. (a) PCoA of bacterial beta-diversity based on the Bray Curtis distance (*p*-value = 0.0003). (b) Multivariate RDA showed significant microbiota among groups (*p*-value = 0.001). Box plots showing alpha diversity; (c) Chao1 richness estimator and (d) Shannon Index. (e) LDA LEfSe plot of taxonomic biomarkers identified in the gut microbiota of different groups at family levels. The LDA score threshold was 3. (f) Boxplots of relative abundance of the phylum.

Microbiota depletion by antibiotic exposure and Se-supplementation had a significant impact on the gut microbiota profile (Adonis with Bray Curtis distance *R*^2^ = 0.246 and *p* = 0.0003) (Figure 4a). This effect was also confirmed by a multivariate RDA (*F* = 2.56 and *p* = 0.001) (Figure 4b).

Se and antibiotic exposure had an impact on the alpha-diversity indexes as microbial diversity (*p* = 0.019, Shannon index) and richness (*p* = 0.006, Chao1 index) (Figure 4c,d). Those differences were not influenced by sequencing coverage as no differences were found between numbers of sequences per group (Figure S1). The Abx mice group showed the lowest microbial diversity and richness compared to the other groups; however, Se-supplementation (Abx-Se) modulated the antibiotic impact in terms of the microbial diversity and richness (*p* < 0.05). No differences in alpha diversity indexes were observed in the CV mice groups with and without Se-supplementation (C–Se *vs* C).

In terms of relative abundances, Se-supplementation and antibiotic-depletion had a relevant impact on microbiota composition (Figures 4 and S2).

No differences were found in the main phyla as Firmicutes, Bacteroides, and Verrucomicrobia between groups. However, other studies have reported significant differences in Firmicutes levels in mice fed a Se-supplemented diet.^40^ Proteobacteria were higher in the C–Se group than in the other groups, showing an increase in this pro-inflammatory phylum^41^ in agreement with other studies that reported a significant increase of its abundance in mice fed Se-enriched *C. megacephalalarvae*.^42^ We also observed an increased abundance of Tenericutes phylum in Se-supplemented groups, both C–Se and Abx-Se, compared to the non-supplemented groups. In agreement with our data, it has been described how higher levels of Tenericutes in beef calves received Se-biofortified alfalfa.^43^ Moreover, Deferribacteres phylum members, concretely *Denitrovibrio acetiphilusN2460*(*T*), have been linked with the capability of growing with dimethyl sulfoxide, selenate, or arsenate provided as a terminal electron acceptor, and 15 genes have been identified that could possibly encode respiratory reductases for these compounds.^44^ In fact, *Deferribacter desulfuricans* has also been reported to grow at the expense of dissimilatory reduction of As(V) to As(III).^45^ In addition to As(V), *D. desulfurican* strain MPA-C3 utilizes NO^3–^, Se(VI), Se(IV), fumarate, and Fe(III) as electron acceptors and acetate, pyruvate, fructose, and benzoate as sources of carbon and energy. In our data, a higher abundance of Deferribacteres members has been identified in Se-supplemented groups, being higher in C–Se than in Abx-Se.

To explore the variation of the microbial community composition between groups, we performed LEfSe tests to detect differences in the relative abundance of bacterial taxa across fecal samples (Figures 4e and S3). LEfSe analysis showed a statistically significant enrichment of the *Deferribacteriaceae*, *Eubacteriaceae*, and *Christensenellaceae* families in the C–Se group, while the *Tannerellaceae* and *Caulobacteraceae* families were enriched in Abx group (Figure 4e). Specifically, the distinction was due to a higher abundance of members of the Deferribacteres phylum in the Se group as compared to the other groups (*p* = 0.001) (Figure 4f).

At the genus level, higher abundances of *Lactobacillus* (*p* = 0.001) and *Flavonifractor* (*p* = 0.002) were observed in CV groups (C and C–Se) and in the Abx-Se group compared to the Abx group (Figure S2). When groups were compared in pairs, the antibiotic treatment induced the reduction of the relative abundance of *Lactobacillus* (*p* < 0.001) and several *Ruminococcaceae* groups, including *Ruminococcaceae_UCG014* (*p* < 0.001) and *Ruminococcaceae_UCG010* (*p* < 0.001) or *Ruminococcaceae_UCG005* (*p* = 0.002) and an enrichment in the *Parabacteroides* genus (*p* < 0.001). However, the supplementation of Se after the antibiotic treatment (Abx *vs* Abx-Se) induced the increase of *Lactobacillus* (*p* < 0.001) and the reduction of the *Parabacteroides* (*p* < 0.001) genus to control levels (no differences in these genera between C and Abx-Se groups).

The genus *Lactobacillus* has been associated with potential beneficial impacts on the host, and most of the *Lactobacillus* species and strains have been considered as probiotic.^46^ The *Lactobacillus* group has been observed in higher abundance in mice groups supplemented with Se, even in the antibiotic microbiota-depleted group. In this regard, it has been shown that the *Lactobacillus* group was increased in diets with median and high Se doses.^42^ In addition, the increase of the *Lactobacillus* genus has also been reported in mice fed a high-fat diet supplemented with Se compared with the un-supplemented group.^40^ Thus, while *Lactobacillus* is significantly reduced in antibiotic-treated mice, the Se-supplementation modulated the impact on the *Lactobacillus* levels in similar levels to control groups (groups Abx-Se and C). In agreement with our data, it has been reported that Se-enriched probiotics (0.3 mg kg^–1^ added to a fermentation medium containing the two probiotic strains of microorganisms, *Lactobacillus acidophilus* and *S. cerevisiae*) affect pig microbiota composition toward an increased abundance of the *Lactobacillus* group and a decrease in *Escherichia coli* abundance.^47^ Another *in vivo* study also reported the effect of Se-containing green tea in the viability and growth of lactic acid bacteria and bifidobacteria.^48^ In the same line, another study reported the positive impact of Se nanoparticles in poultry feed on the levels of potential beneficial bacteria such as *Faecalibacterium prausnitzii*, *Lactobacillus* spp., and *Ruminococcus* spp. as well as the total short-chain fatty acids (SCFAs), in particular the increase of butyric acid.^49^*Ruminococcaceae_UCG014* has been linked with Se-yeast-supplemented laying hens and it is a common family related with the maintenance of gut health and had the enzymatic ability to degrade cellulose and hemicellulose.^50^ Another study also reported the impact of supplementation of inorganic Se in dogs on the enrichment of family *Ruminococcaceae*, including the genera *Catenibacterium*, *Holdemanella*, and *Ruminococcaceae UCG-014*, and also organic Se increased the presence of the *Lactobacillus* genus and decreased the presence of *E. coli* (Proteobacteria phylum).^51^ Our results showed an increase in *Christensenellaceae* members on the Se group of CV mice. This microbial group has been described as a highly heritable microbe in humans and it has been also associated with health^52^ and inversely related to host body mass index in different populations and multiple studies. Although we observed that Se-supplementation increased the relative abundance of the *Christensenellaceae* group, antibiotics caused a dramatic reduction of these bacteria, which cannot be modulated by Se. Studies using GF mice fed Se-supplementation (0.4 mg Se kg^–1^) showed a potential beneficial impact on microbial diversity^13^ in a manner similar to CV. Thus, the potential effect of Se-supplementation on the gut microbiota modulation has been suggested. Although the mechanisms by which Se shape gut microbiota bacteria are complex, we have reported the potential benefit in the gut microbiota even when microbiota were depleted with antibiotics groups. Our observations highlight three important results: (i) significant stimulation of potential beneficial bacteria such as *Lactobacillus, Ruminococcaceae*, and *Christensenellaceae* members, (ii) significant increase in microbial diversity and (iii) richness. Further studies would be necessary to understand the exact mechanisms of microbiota–Se interactions and the potential benefits for health.

Furthermore, despite the evidence on the impact of Se or specific enriched Se-foods on specific microbial groups,^8,13,53^ little is known about the impact on the selenoproteome profile.

### Associations between Gut Microbiota and Selenoproteomes in Plasma

**Figure 5:**
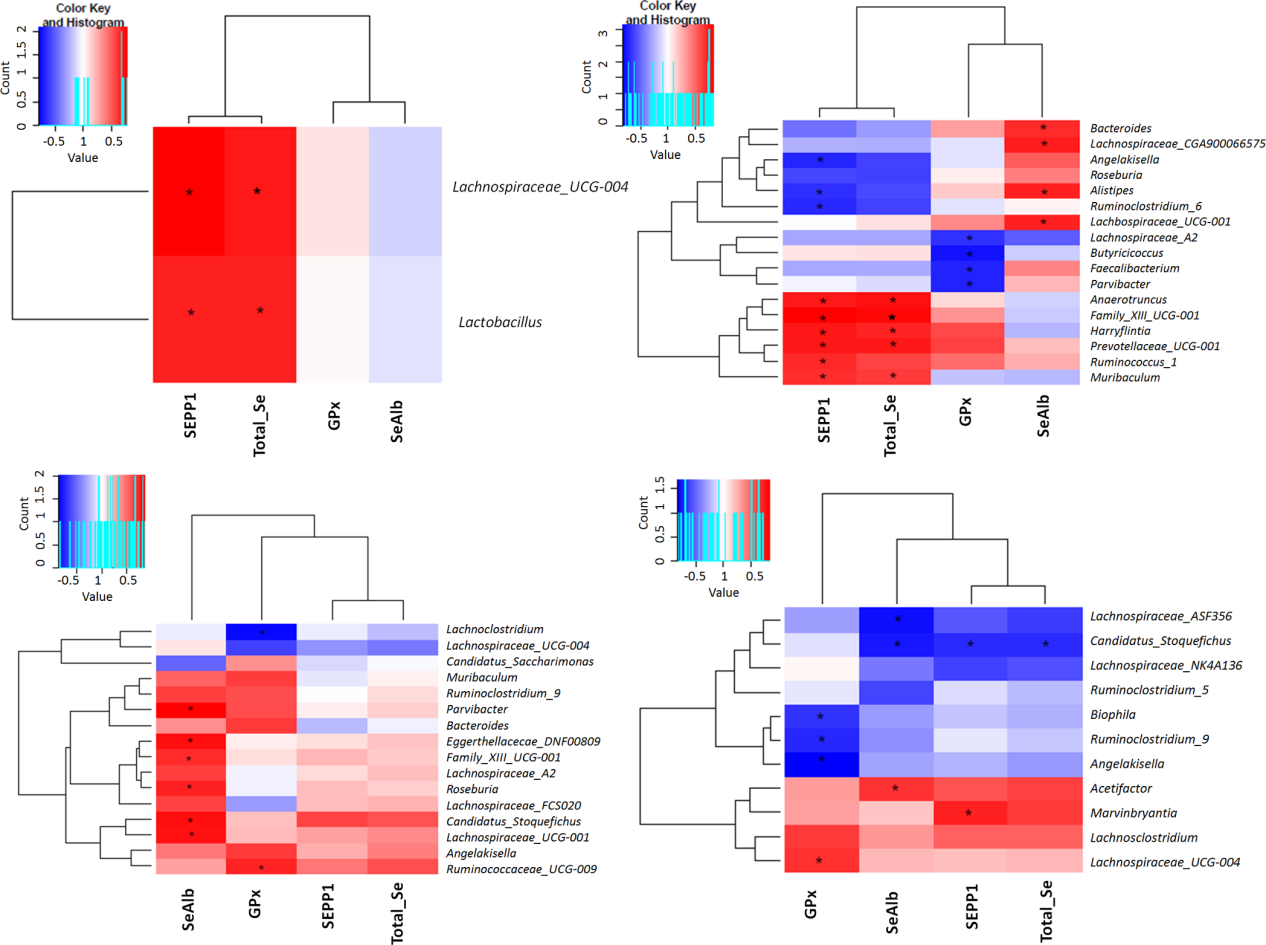
Spearman correlation matrix heatmaps for mice plasma selenoproteins and gut microbiota genus in the (a) Abx group, (b) Abx-Se group, (c) C group, and (d) C–Se group.

As potential links between gut microbiota metabolism and the plasma selenoproteome, we investigated the potential associations between gut microbial taxa and the plasma selenoproteome profile in the studied groups C, C–Se, Abx, and Abx-Se (Table S6). A significant reduced number of associations were observed in the microbiota-depleted mice (Abx) explained by the reduction in microbial diversity and richness. In this sense, only higher *Lactobacillus* (rho = 0.71, *p* = 0.03) and *Lachnospiraceae_UCG-01* (rho = 0.79, *p* = 0.03) genus were associated with higher SEPP1 in Abx (Figure 5a). On the contrary, the number of correlations between bacteria and selenoproteins increased significantly after Se supplementation in Abx-Se (Figure 5b), suggesting again the intertwined mechanism between Se and microbiota. Interestingly, higher SEPP1 was associated with a lower abundance of *Alistipes* (rho = −0.7, *p* = 0.03) and *Ruminoclostridium_6* (rho = −0.71, *p* = 0.02) genus, and a higher abundance of *Anaerotruncus* (rho = 0.77, *p* = 0.01), *Angekisella* (rho = −0.68, *p* = 0.03), *Family_XII_UCG-001* (rho = 0.85, *p* = 0.002), *Prevotellaceae_UCG-001* (rho = 0.77, *p* = 0.01), and *Ruminococcus_1* (rho = 0.85, *p* = 0.002) genus. On the other hand, the concentration of GPx was positively correlated with *Parvibacter* (rho = 0.67, *p* = 0.05) and *Ruminococcaceae_UCG-009* (rho = 0.74, *p* = 0.02) and negatively with *Lachnospiraceae_UCG-004* (rho = −0.68, *p* = 0.04) in the C group (Figure 5c). No significant correlations of these bacteria with GPx were found in mice with depletion of microbiota (Abx). However, *Parvibacter* genus (rho = −0.72, *p* = 0.02) were inversely associated with this selenoprotein in mice fed a Se-supplemented diet (C–Se) and microbiota-depleted mice fed a Se-supplemented diet (Abx-Se), respectively. In the same way, positive correlations between SeAlb and *Lachnospiraceae_UCG-001* were observed in C (rho = 0.82, *p* = 0.02) and Abx-Se (rho = 0.73, *p* = 0.02), but no associations in Abx were found. Finally, in terms of diversity, only the Abx-Se group showed significant associations. In this group, GPx was positively correlated with the Shannon index (rho = 0.69, *p* = 0.029) and SeAlb with the Chao1 index (*R* = 0.66, *p* = 0.038).

### Associations between Trace Elements Homeostasis in Plasma and Gut Microbiota According to Se-Supplementation and Antibiotic–Microbiota Disruption

A correlation analysis between total elements in plasma and genus was reported for the first time. Our results showed that Al, Co, Cu, Mn, V, and Zn correlated with different genera in C, C–Se, Abx, and Abx-Se (Table S7). Per groups, Abx-Se showed the highest number of associations with genus (11 significant correlations) especially with Al, followed by C and C–Se, which presented a total of seven significant correlations per group. However, no correlations between elements and genera were found in the Abx group. This fact may indicate the intertwined role of Se and gut microbiota in metal homeostasis, which is in good agreement with the previously discussed results. In this sense, *Enterorhabdus* (rho = −0.88, *p* = 0.01), *Erysipelatoclostridium* (rho = −0.63, *p* = 0.04), and *Ruminococcaceae_UCG-010* (rho = −0.82, *p* = 0.02) were negatively associated with Al in the Abx-Se group. Moreover, higher *Flavonifractor* (rho = 0.78, *p* = 0.01) and *Ruminiclostridium_9* (rho = 0.75, *p* = 0.02) were associated with higher Al. In addition, we found that higher *Subdoligranulum* (rho = 0.71, *p* = 0.02) correlated with higher V in the same group. In the C–Se group, *Prevotellaceae_UCG-001* (rho = 0.85, *p* = 0.03) and *Ruminiclostridium* (rho = 0.87, *p* = 0.01) were positively correlated with Mn. Finally, we observed that Co was positively associated with *Acetobacter* in C–Se but negatively in Abx-Se.

In summary, we can conclude that plasma selenoproteome and metal homeostasis were considerably affected by Se-supplementation, possibly by the interplay between Se and gut microbiota. Our study demonstrated the potential beneficial effects of Se on the gut microbiota, especially after microbiota depletion by antibiotics as well as the associations of specific bacteria with plasma selenoproteins GPx, SEPP1, and SeAlb and the concentrations of some elements. However, further studies are needed to identify the specific Se–microbiota interactions and the potential implication in health outcomes.

## Supporting Information Available

Reproducibility of the analysis using human serum BCR-637 CRM; reproducibility of the analysis ICP-QQQ-MS using ClinChek, Serum Level II as control for trace elements; relative abundances at the phylum level for each group; relative abundances at the family level for each group; relative abundances at the genus level for each group; Spearman correlation coefficients between metals and genus; Spearman correlation coefficients between selenoproteins and genus; number of sequences at the (a) group level and the (b) individual level; Boxplot of abundance corresponding to genera with significant differences in Wilcoxon test with FDR; and LDA effect size plot of taxonomic biomarkers identified in the gut microbiota of different groups at genus levels and compared between groups (PDF)

## Abbreviationspara

Abx: antibiotic-treated mice fed a rodent diet
Abx-Se: antibiotic-treated mice fed a Se-supplementation diet
AF: affinity chromatography
C: control mice fed a rodent diet
C–Se: mice fed a Se-supplementation diet
CV: conventional
GF: germ-free
GPx: plasma glutathione peroxidase
ICP-QQQ-MS: triple quadrupole inductively coupled plasma mass spectrometry
LDA: linear discriminant analysis
LEfSe: effect size (LEfSe) plot
PCoA: principal coordinate analysis
RDA: redundancy discriminant analysis
SeAlb: selenoalbumine
SEC: size exclusion chromatography
SEPP1: selenoprotein P
