# Supplementary material for: Impact of Antibiotic-Induced Depletion of Gut Microbiota and Selenium Supplementation on Plasma Selenoproteome and Metal Homeostasis in a Mice Model
Source: J Agric Food Chem. 2021 Jun 25;69(27):7652–62. doi: 10.1021/acs.jafc.1c02622 (PMC9161447; doi:10.1021/acs.jafc.1c02622)
Supplement: Supplementary file 1 — jf1c02622_si_001.pdf [file jf1c02622_si_001.pdf]

Impact of antibiotic-induced depletion of gut microbiota and selenium supplementation on plasma selenoproteome and metal homeostasis in mice model

Belén Callejón-Leblic[a], Marta Selma-Royo[b], María Carmen Collado[bΣ], Nieves Abril[cΣ], Tamara García-Barrera[aΣ*]

[a]Research Center of Natural Resources, Health and the Environment (RENSMA). Department of Chemistry, Faculty of Experimental Sciences, University of Huelva, Fuerzas Armadas Ave., 21007, Huelva, Spain; [b]Institute of Agrochemistry and Food Technology-National Research Council (IATA-CSIC), Department of Biotechnology, Agustin Escardino 7. 46980 Paterna, Valencia, Spain, [c]Department of Biochemistry and Molecular Biology, University of Córdoba, Campus de Rabanales, Edificio Severo Ochoa, E-14071, Córdoba, Spain. Σ senior authors; *tamara@dqcm.uhu.es

Selenium species	Human Serum BCR-637 (SEC-AF-ICP-MS) n=5		Human Serum BCR-637 + 50 ng of Se of selenite per g of serum (SEC-AF-ICP-MS) n=5		Human Serum BCR-637[a] (AE-SPE/AF-ICP-MS) n=5		LD ($ng\ g^{-1}$ of Se)
	Average concentration ($ng\ g^{-1}$)	Reproducibility %RSD	Average concentration ($ng\ g^{-1}$)	Reproducibility %RSD	Average concentration ($ng\ g^{-1}$)	Reproducibility %RSD	
GPx	11	2	11	3	15	4	0.5
Selenometabolites	-	-	46	4	-	-	0.5
SEPP1	59	3	61	4	60	7	0.5
SeAlb	15	3	16	3	13	4	0.5
Total Se	85	4	132	5	90	7	0.5

Table S1. Reproducibility of the analysis using Human Serum BCR-637 certified reference material. [a]Concentration of selenoproteins determined by Jitaru et al[1] using 13 different methodologies. SEC: size exclusion chromatography, AF: affinity chromatography, AE: anion exchange chromatography, SPE: solid phase extraction. LD: Limit of detection. Certified concentration of Se in Human Serum BCR-63 reference material = 81 ±7 $ng\ ml^{-1}$.

[1] Jitaru, P., Roman, M., Barbante, C. et al. Challenges in the accurate speciation analysis of selenium in humans: first report on indicative levels of selenoproteins in a serum certified reference material for total selenium (BCR-637). Accred Qual Assur 15, 343–350 (2010). https://doi.org/10.1007/s00769-010-0637-1

Elements	Clinchek Serum Level II, n=5			
	Average concentration (ng g^{-1})	Reproducibility RSD %	Certified concentration (ng g^{-1})	LOD (ng g^{-1})
Al	50.15	10	43-64.5	0.06
V	8.23	7	6.47-9.71	0.04
Cr	6.73	9	4.53-6.79	0.04
Mn	4.96	8	4.83-7.24	0.04
Fe	1516.58	8	1190-1780	0.05
Co	5.72	3	4.59-5.89	0.05
Ni	5.69	8	4.77-7.15	0.04
Cu	1440.99	9	1150-1560	0.05
Zn	1629.64	6	1280-1740	2.97
As	23.67	5	14.5-24.1	0.01
Se	120.87	6	89.8-135	0.03
Mo	5.55	10	4.33-6.5	0.04
Cd	6.1	10	4.04-6.73	0.04
Sb	7.39	6	5.64-8.46	0.04
Tl	6.2	8	5.59-8.38	0.04
Pb	8.02	10	Non certified	0.04

Table S2. Reproducibility of the elemental analysis using Clinchek Serum Level II Control for trace elements.

Phylum	C	C-Se	Abx	Abx-Se	p-value
Patescibacteria	0.16	0.11	0	0	<0.001
Deferribacteres	0.05	0.53	0	0	0.001
Tenericutes	0.01	0.02	0	0.15	0.004
Proteobacteria	0.63	0.74	0.58	0.16	0.010
Actinobacteria	1.37	1.34	1.1	1.08	0.89
Verrucomicrobia	0.01	0.015	0.015	0.015	0.89
Bacteroidetes	10.36	15.27	20.21	16.3	0.89
Firmicutes	88.03	80.42	77.27	80.95	0.89

Table S3. Relative abundances at phylum level for each group. P-value from Wilcoxon test with False Discovery test Rate (FDR).

Family	C	C-Se	Abx	Abx-Se	p-value
Saccharimonadaceae	0.16	0.11	0	0	<0.001
Christensenellaceae	0.01	0.055	0	0.03	0.001
Eubacteriaceae	0.01	0.025	0	0	0.001
Deferribacteraceae	0.05	0.53	0	0	0.001
Anaeroplasmataceae	0	0.01	0	0.14	0.001
Lactobacillaceae	1.52	1.84	0.005	1.14	0.001
Marinifilaceae	0.76	0.98	0	0	0.002
Tannerellaceae	0.06	0.085	0.74	0.03	0.003
Desulfovibrionaceae	0.63	0.46	0.34	0	0.008
Staphylococcaceae	0.02	0.045	0	0	0.010
Streptococcaceae	0.23	0.65	0.12	0.27	0.023
Clostridiales_vadinBB60_group	0	0.06	0.025	0.16	0.029
Erysipelotrichaceae	0.2	0.62	0.58	1.42	0.045

Table S4. Relative abundances at family level for each group. P-value from Wilcoxon test with False Discovery test Rate (FDR).

Genus	C	C-Se	Abx	Abx-Se	p-value
Lachnospiraceae_GCA900066225	0.05	0.14	0	0	<0.001
Candidatus_Saccharimonas	0.16	0.11	0	0	<0.001
Anaerofustis	0.01	0.025	0	0	0.001
Anaeroplasma	0	0.01	0	0.14	0.001
Erysipelatoclostridium	0	0	0.17	0.12	0.001
Harryflintia	0	0.03	0	0.005	0.001
Lactobacillus	1.52	1.84	0.005	1.14	0.001
Mucispirillum	0.05	0.53	0	0	0.001
Ruminococcaceae_UCG005	0.05	0.03	0	0	0.001
Ruminococcaceae_UCG010	0.07	0.085	0	0	0.001
Ruminococcaceae_UCG014	0.66	0.54	0.015	0.2	0.001
Odoribacter	0.76	0.98	0	0	0.002
Flavonifractor	0.27	0.1	0.04	0.04	0.002
Lachnospiraceae_A2	0.04	1.39	4.55	5.29	0.003
Ruminococcaceae_UCG003	0.15	0.08	0	0	0.003
Peptococcus	0.03	0.055	0	0	0.003
Parabacteroides	0.06	0.085	0.74	0.03	0.003
Butyricicoccus	0.45	0.55	0.07	0.08	0.004
Ruminiclostridium_5	0.37	0.86	0.16	0.33	0.006
Parvibacter	0.07	0.03	0	0	0.007
Candidatus_Stoquefichus	0.01	0.01	0.01	0	0.009
Staphylococcus	0.02	0.045	0	0	0.009
Unclassified	29.37	35.72	26.34	36.59	0.009
Ruminiclostridium	0.19	0.42	0.05	0.055	0.011
Ruminiclostridium_6	0.66	0.26	0	0.075	0.011
Ruminococcaceae_UCG009	0.01	0.025	0	0	0.012
Lachnospiraceae_GCA900066575	0.29	0.44	0.42	0.16	0.013
Lachnospiraceae_UCG004	0.12	0.13	0	0.18	0.019
Streptococcus	0.23	0.65	0.12	0.27	0.019
Acetatifactor	0.35	0.5	0.31	0.015	0.028
Ruminococcus_1	0.22	0.03	0	0.005	0.028
Ruminococcaceae_NK4A214_group	0.04	0.03	0	0	0.035

Table S5. Relative abundances at genus level for each group. P-value from Wilcoxon test with False Discovery test Rate (FDR).

Selenoproteins	Genus	Spearman Correlation Coefficient and *abundance %			
		C	C-Se	Abx	Abx-Se
eGPx	*Bacteroides*	0.67 *(1.4%)	N.S	N.S	N.S
	Bilophila	N.S	-0.66*(0.5%)	N.S	N.S
	Butyricicoccus	N.S	N.S	N.S	-0.79*(0.3%)
	Lachnoclostridium	-0.83 *(7%)	N.S	N.S	N.S
	Lachnospiraceae_A2	N.S	N.S	N.S	-0.68 *(7%)
	Lachnospiraceae_UCG-004	-0.68 *(0.1%)	N.S	N.S	N.S
	***Parvibacter*	0.67*(0.1%)	N.S	N.S	-0.72*(0.02%)
	Prevotellaceae_UCG-001	N.S	N.S	N.S	0.63*(0.02%)
	Ruminococcaceae_UCG-009	0.74*(0.03%)	N.S	N.S	N.S
SEPP1	*Alistipes*	N.S	N.S	N.S	-0.7*(1.4%)
	Anaerotruncus	N.S	N.S	N.S	0.77 *(0.7%)
	Angelakisella	N.S	N.S	N.S	-0.68*(0.05%)
	Family_XIII_UCG-001	N.S	N.S	N.S	0.85*(0.2%)
	Harryflintia	N.S	N.S	N.S	0.8*(0.01%)
	Lachnospiraceae_UCG-004	N.S	N.S	0.79*(1.5%)	N.S
	Lactobacillus	N.S	N.S	0.71*(0.01%)	N.S
	Marvinbryantia	N.S	0.71*(0.2%)	N.S	N.S
	Prevotellaceae_UCG-001	N.S	N.S	N.S	0.77*(0.02%)
	Ruminiclostridium_6	N.S	N.S	N.S	-0.71*(0.2%)
	Ruminococcus_1	N.S	N.S	N.S	0.85*(0.1%)
SeAlb	***Lachnospiraceae_UCG-001*	0.82*(3%)	N.S	N.S	0.73*(0.7%)
	Alistipes	N.S	N.S	N.S	0.73*(1.4%)
	Candidatus_Stoquefichus	0.9*(0.01%)	N.S	N.S	N.S
	DNF00809	0.78*(0.3%)	N.S	N.S	N.S
	GCA-900066575	N.S	N.S	N.S	0.73*(0.2%)
	Parvibacter	0.85*(0.1%)	N.S	N.S	N.S
Total Se	*ASF356*	N.S	-0.64* (0.02%)	N.S	N.S
	Family_XIII_UCG-001	N.S	N.S	N.S	0.83 *(0.01%)
	Harryflintia	N.S	N.S	N.S	0.76*(0.01%)
	Lachnospiraceae_UCG-004	N.S	N.S	0.71*(0.05%)	N.S
	Prevotellaceae_UCG-001	N.S	N.S	N.S	0.77*(0.02%)
	Ruminococcus_1	N.S	N.S	N.S	0.77*(0.1%)

Table S6. Spearman correlation coefficients between selenoproteins and genus. Only significant correlation coefficients ($p<0.05$) are shown in the table. **Genus correlated with selenoproteins in two groups at least. N.S.: Non-significant.

Elements	Genus	Correlation Coefficient and *abundance %			
		C	C-Se	Abx	Abx-Se
Al	*Enterorhabdus*	N.S	N.S	N.S	-0.88 *(0.9%)
	Erysipelatoclostridium	N.S	N.S	N.S	-0.63 *(0.6%)
	Flavonifractor	N.S	N.S	N.S	0.78 *(0.4%)
	Ruminiclostridium_9	N.S	N.S	N.S	0.75 *(0.8%)
	Ruminococcaceae_UCG-010	N.S	N.S	N.S	-0.82 *(0.03%)
	Angelakisella	-0.76 *(0.03%)	N.S	N.S	N.S
	Lachnospiraceae_NK4A136_group	0.71*(34%)	N.S	N.S	N.S
	Clostridiales_vadinBB60_group	N.S	0.77*(0.03%)	N.S	N.S
Co	***Acetatifactor*	N.S	0.68*(0.9%)	N.S	-0.66 *(0.2%)
	Ruminiclostridium_6	N.S	N.S	N.S	-0.73 *(0.2%)
	Clostridiales_vadinBB60_group	0.91*(0.03%)	N.S	N.S	N.S
	Ruminococcaceae_UCG-005	-0.7 *(0.4 %)	N.S	N.S	N.S
	Candidatus_Stoquefichus	N.S	-0.9 *(0.01%)	N.S	N.S
Cu	*Intestinimonas*	N.S	N.S	N.S	-0.68 *(0.6%)
	Lachnospiraceae_UCG-001	N.S	N.S	N.S	-0.88 *(0.7%)
	Caulobacter	N.S	-0.75 *(0.01%)	N.S	N.S
	Ruminococcaceae_UCG-002	N.S	-0.78 *(0.01%)	N.S	N.S
Mn	*Prevotellaceae_UCG-001*	N.S	0.85 *(0.2%)	N.S	N.S
	Ruminiclostridium	N.S	0.87 *(0.6%)	N.S	N.S
	Lactobacillus	0.85 *(2%)	N.S	N.S	N.S
V	*Subdoligranulum*	N.S	N.S	N.S	0.71*(0.02%)
Zn	*Lachnospiraceae_UCG-001*	N.S	N.S	N.S	-0.75*(0.7%)
	Angelakisella	-0.79*(0.03%)	N.S	N.S	N.S
	Lactobacillus	0.67 *(2%)	N.S	N.S	N.S

Table S7. Spearman correlation coefficients between metals and genus. Only significant correlation coefficients (p>0.05) are shown in the table. **Genus correlated with the same element in two groups at least. N.S.: Non-significant.

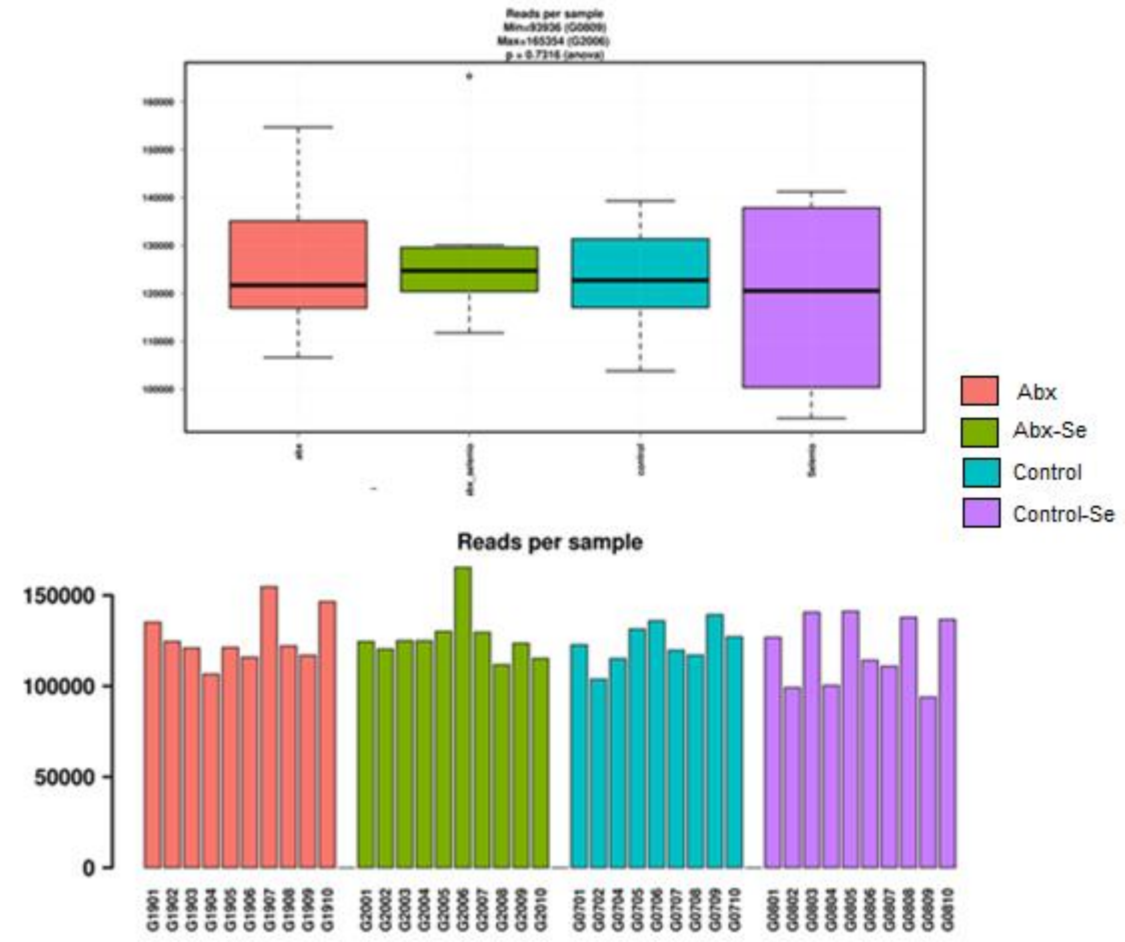

Figure S1.Number of sequences at a) group level and b) individual level. No differences in the sequencing coverage were observed between groups

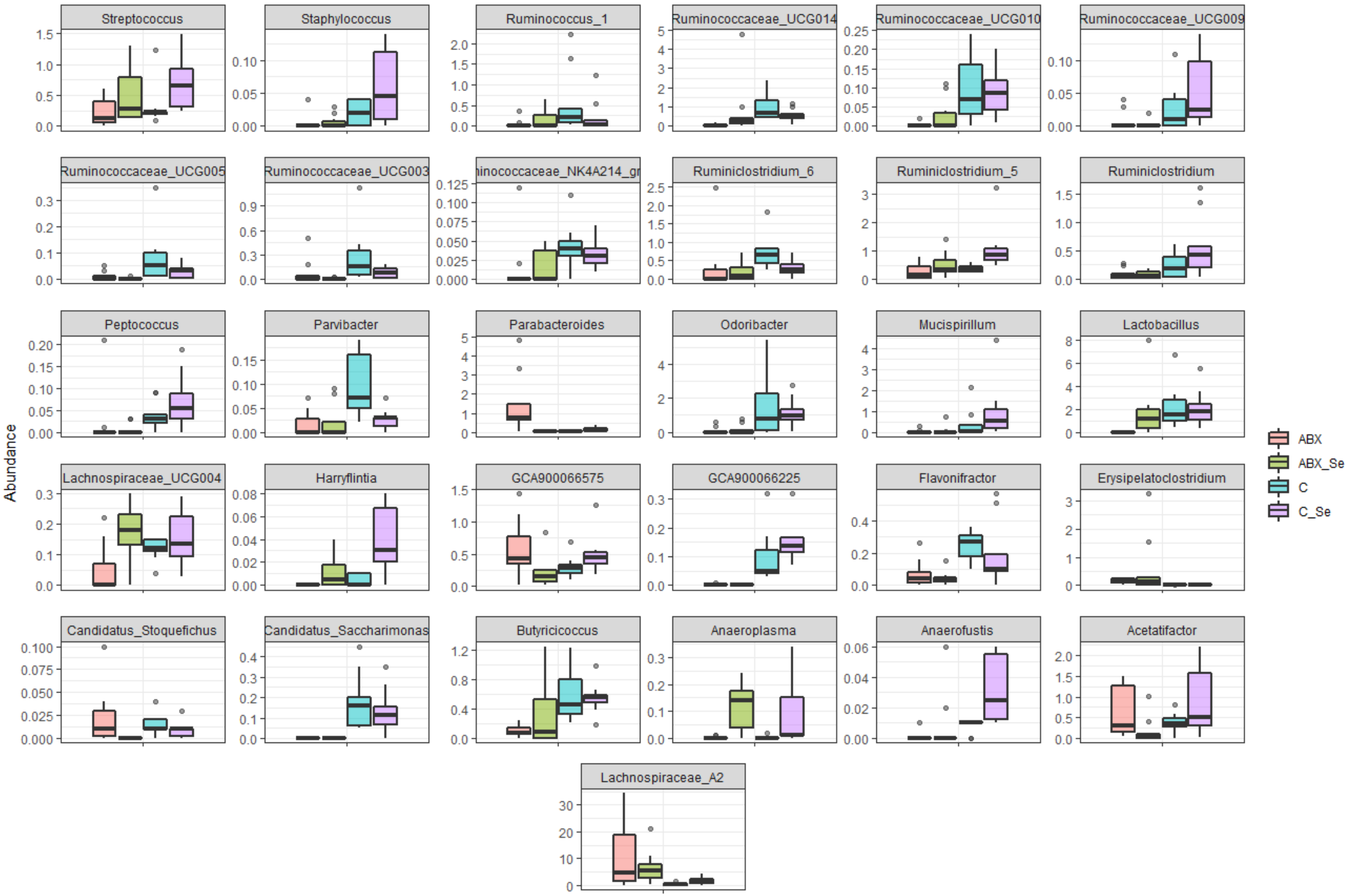

Figure S2.Boxplot of abundance corresponding to genera with significant differences in Wilcoxon test with False Discovery test Rate (FDR).

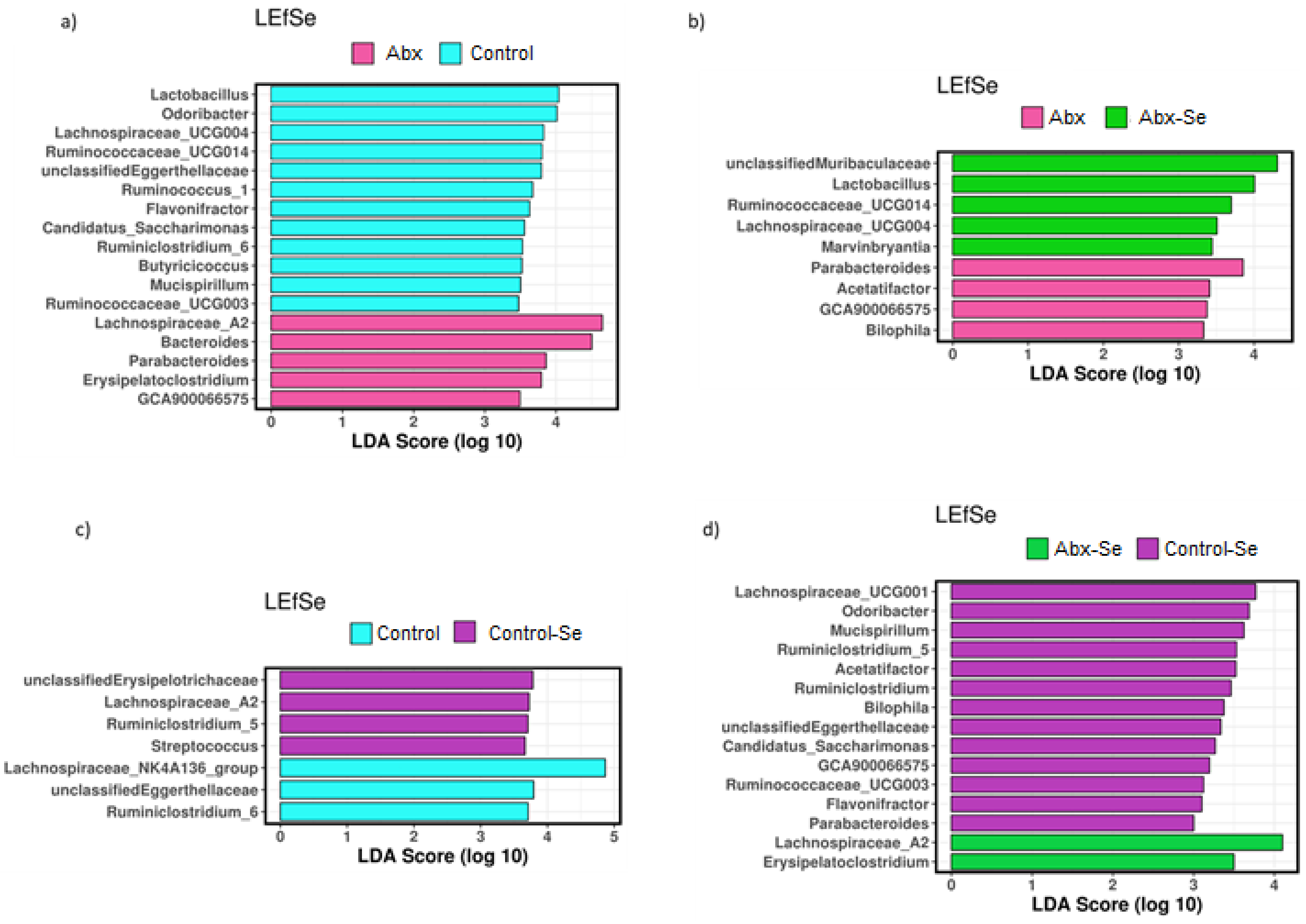

Figure S3. Linear Discriminant Analysis (LDA) Effect Size (LEfSe) plot of taxonomic biomarkers identified in the gut microbiota of different groups at genus levels and compared between groups. The LEfSe algorithm, emphasizing both statistical and biological relevance, was used for biomarker discovery. The threshold for the logarithmic discriminant analysis (LDA) score was 3.0
